# Municipal Characteristics of In-Home Death Among Care-Dependent Older Japanese Adults

**DOI:** 10.1001/jamanetworkopen.2021.42273

**Published:** 2022-01-05

**Authors:** Kazuhiro Abe, Ichiro Kawachi, Yuta Taniguchi, Nanako Tamiya

**Affiliations:** 1 Takemi Program in International Health, Harvard T.H. Chan School of Public Health, Boston, Massachusetts; 2 Department of Public Health, Graduate School of Medicine, The University of Tokyo, Tokyo, Japan; 3 Department of Health Services Research, Faculty of Medicine, University of Tsukuba, Ibaraki, Japan; 4 Department of Social and Behavioral Sciences, Harvard T.H. Chan School of Public Health, Boston, Massachusetts; 5 Graduate School of Comprehensive Human Sciences, University of Tsukuba, Ibaraki, Japan; 6 Health Services Research and Development Center, University of Tsukuba, Ibaraki, Japan

## Abstract

**Importance:**

The provision of in-home and community end-of-life care has emerged as an important policy issue for aging populations around the world. Despite most patients expressing the wish to die at home (as opposed to in the hospital), substantial geographic variation persists in the prevalence of in-home death at the end of life.

**Objective:**

To assess the association of municipal characteristics with variation in the place of death among care-dependent older people in Japan.

**Design, Setting, and Participants:**

In this cross-sectional study using multilevel logistic regression, analyses were performed using national claims data under Japan’s long-term care insurance system. The study population included long-term care insurance beneficiaries 65 years and older who died in 2015, excluding those who died from external causes, such as unintentional injuries. Data analyses were conducted from January 1 to April 31, 2021.

**Exposures:**

Predisposing, enabling, and need factors at the individual and municipal levels according to a behavioral model.

**Main Outcomes and Measures:**

The outcome was whether individuals died at home or not.

**Results:**

This cross-sectional study analyzed 544 836 decedents (median [IQR] age, 87 [81-91] years; 300 142 [55.1%] female). The proportion of in-home deaths was 10.3%. In the multilevel null model, 7.2% of the variance in the place of death was associated with municipal-level characteristics. Municipal characteristics were associated with more of the variance than were prefectural characteristics (2.7%). The largest proportional change (7.3%) in variance at the municipality level was observed when enabling factors (ie, medical and long-term care resources) were added to the model. Municipalities with a high proportion of in-home death were more likely to have a higher supply of clinics, physicians, and care workers providing in-home services per population, as well as a lower number of hospital beds and long-term care facility workers per population.

**Conclusions and Relevance:**

In this cross-sectional study, municipal characteristics explained a substantial proportion of the geographic variance in in-home death in Japan. These results suggest that municipal policy makers need to ensure an adequate supply of clinics, physicians, and care workers providing in-home services to meet the preferences of care-dependent older people who wish to spend their final days at home.

## Key Points

**Question:**

To what extent can municipal characteristics explain geographic variation in the ability of care-dependent older people to stay at home at the end of life?

**Findings:**

This cross-sectional study with multilevel logistic analysis of 544 836 deaths found that 7.2% of the variance in place of death at the end of life was associated with municipal characteristics. In particular, higher accessibility to in-home services provided by physicians, nurses, and care workers at the municipal level was associated with a higher probability of in-home death.

**Meaning:**

These results suggest that policy makers at the municipal level should ensure an adequate supply of clinics, physicians, and care workers providing in-home services to meet the preferences of care-dependent older people who wish to spend their final days at home.

## Teaser

This cross-sectional study assesses whether municipal characteristics are associated with in-home deaths vs deaths outside the home among older Japanese adults.

## Introduction

As societies throughout the world grapple with population aging, an important issue that has emerged is how to ensure the quality of end-of-life care. The quality of dying and death would be partially determined by the degree to which a person’s preferences for the location of death (eg, in the hospital vs in the home) are fulfilled.^1,2,3,4,5^ Generally, older adults prefer to die at home surrounded by their loved ones.^6,7^ In turn, the satisfaction of informal caregivers (ie, family) with terminal caregiving is higher when the wishes of care-dependent older people at the end of life are fulfilled.^8^ However, even though 55% of the Japanese people 55 years and older express the wish to spend the end of their life at home,^9^ the country has a low proportion of in-home death (13.2% in 2017) compared with Canada (59.9%), England (46.0%), and the US (30.7%).^10,11^ Thus, it is important from a policy perspective to understand what can be done to bridge the gap between people’s wishes and reality at the end of life.

In previous studies,^2,5,12,13,14,15,16,17,18,19^ both individual and regional factors associated with the place of death have been reported. At the individual level, in-home deaths have been associated with personal preferences for in-home death; older age; female sex; low functional status; cancer diagnoses or nonacute diseases; and a history of use of in-home services, day services, and short-stay services at the end of life, as well as the presence of informal caregivers.^2,5,12,13^ In ecological studies,^2,12,14,15,16,17,18,19^ in-home death has been associated with higher access to clinics or agencies delivering in-home services provided by physicians, nurses, and care workers, as well as day services provided by care workers. By contrast, numbers of hospitals and nursing home beds per population have been inversely correlated with in-home death.^2,12,15,16,17,18^ Furthermore, substantial geographic variation persists in the proportion of in-home deaths across Japanese municipalities, according to the mapping by Morioka et al.^16^

In Japan, local governments are organized at 2 levels: prefectures and municipalities. Municipalities are primarily responsible for the planning and delivery of long-term care (LTC).

Care-dependent older people in Japan are eligible to receive medical and LTC services provided under the universal health insurance and LTC insurance system. The health insurance covers inpatient care, outpatient care, in-home medical care, and palliative care provided mainly by physicians and nurses. Copayments range from 10% to 30% of the total medical cost, depending on patient’s age and income. On the other hand, LTC insurance covers LTC services for home-dwelling recipients (ie, in-home services, day services, and short-stay services) and for residents at LTC facilities provided mainly by care workers.^13^ When an older person who needs to use LTC services applies to the municipality, medical and welfare professionals determine the level of care needed (categorized into 7 levels), considering the results from an in-home assessment by municipal assessors and the opinion of the primary physician. The 7 levels include support levels 1 and 2, indicating the need for support of instrumental activities of daily living, and care levels 1 to 5, requiring help in performing activities of daily living. A higher level corresponds to higher requirements for care.^20^ The level of care needed stipulates the maximum amount to be covered by LTC insurance. Copayments range from 10% to 30% of LTC cost, depending on recipient’s income. For more information on the LTC services provided in Japan, we refer readers to the description provided by Abe et al.^13^

Given the low proportion of in-home deaths in Japan compared with other countries and patient preferences, we sought to understand the municipal characteristics that could explain the variation in place of death among older persons at the end of life to guide the allocation of resources to support people who prefer to die at home.

## Methods

### Study Design and Population

We conducted a cross-sectional, 3-level study. The study population (level 1) included LTC insurance beneficiaries, 65 years and older, who died in 2015, excluding those who died by external causes, such as unintentional injuries and suicides (codes V01-Y89 in* International Statistical Classification of Diseases and Related Health Problems, Tenth Revision *[*ICD-10*]). Individuals were nested within 1577 municipalities (level 2), which were in turn nested within 47 prefectures (level 3). Municipalities are further divided into cities, towns, and villages according to population size and are responsible for the delivery of many services, including LTC. Prefectures are responsible for administration over a wider area than municipalities. Data analyses were conducted from January 1 to April 31, 2021. All data were anonymized by the Japanese Ministry of Health, Labour, and Welfare and then provided to us. In accordance with the Ethical Guidelines for Medical and Biological Research Involving Human Subjects published by the Japanese government, informed consent was waived. This research was performed with approval from the ethics review committees of the University of Tokyo and the University of Tsukuba. This study followed the Strengthening the Reporting of Observational Studies in Epidemiology (STROBE) reporting guideline for cross-sectional studies.

### Data Sources

We used individual-level administrative data, including linkage of data from the Statistics of Long-term Care Benefit Expenditures, death records from the Vital Statistics, Survey of Medical Institutions, and Survey of Institutions and Establishments for Long-term Care with official approval from the Japanese Ministry of Health, Labour, and Welfare.^21^ The Japanese Ministry of Health, Labour, and Welfare anonymized these data. For municipal characteristics, we used aggregated data from the Population Census, Statistics of Physicians, Dentists, and Pharmacists, Comprehensive Survey of Living Conditions, statistical reports on land areas, annual statistics on local public finance, survey of municipal taxation, and life tables, all published by the Japanese government.^22^ The eTable in the Supplement provides the data sources for each variable.

We performed deterministic linkage between the Vital Statistics death records and the Statistics of Long-term Care Benefit Expenditures based on the individual’s municipality of residence, sex, month and year of birth, and date of death.^13^ The merged data linked decedents using anonymized identifiers that do not change unless one moves out of the municipality. Approximately 0.3% of those who died according to the death records were excluded because of the lack of exact date of birth. We extracted 575 589 LTC insurance beneficiaries who were 65 years and older and died in 2015, excluding external causes of death. Among them, 30 753 decedents were excluded because of the missing data on marital status, care levels, and municipal variables.

### Outcome and Explanatory Variables

The outcome was whether individuals died at home or not. Other places of death included hospitals, clinics, LTC facilities, and other locations, such as day services facilities or outdoors. The place of death was determined from the Vital Statistics death records, which were created from the death certificate written by the physician who certified the death.

We used a behavioral model of health services utilization (6th revision) created by Andersen et al^23^ to classify individual and contextual factors according to their theoretical linkage to in-home death. The explanatory variables (classified according to *predisposing, enabling*, and *need* factors) are summarized in the Figure. Individual characteristics included the patients’ age at death, sex, level of care needed, the most common underlying causes of death in the Japanese population 65 years and older (ie, cancer [*ICD-10* codes C00-C97], cerebrovascular diseases [*ICD-10* codes I60–I69], cardiovascular diseases [*ICD-10* codes I01-I02.0, I05-I09, I20-I25, I27, and I30-I52], senility [*ICD-10* code R54], and pneumonia [*ICD-10* codes J12-J18]), and marital status (ie, present, unmarried, widow, or divorce).

**Figure.**
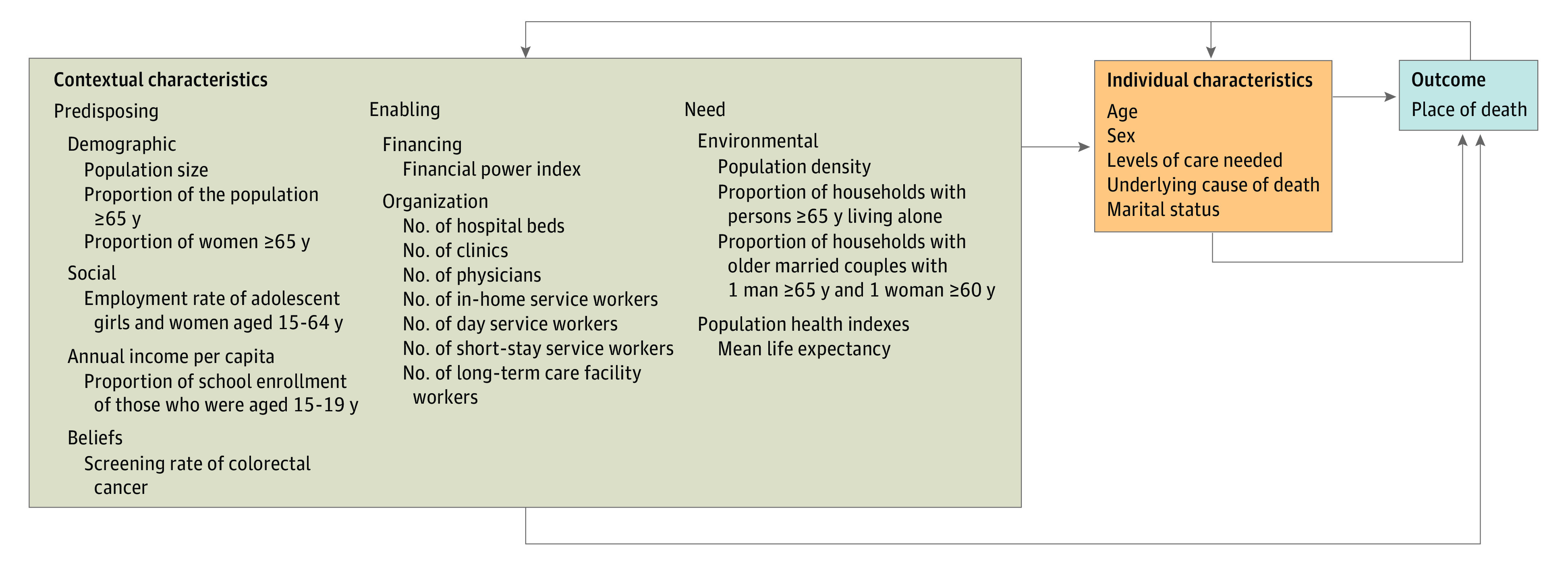
Classification of Explanatory Variables Based on the Behavioral Model (6th Revision) of Andersen et al^23^ The financial power index of each municipality was calculated by the 3-year mean of base financial revenue divided by base financial demand. The number of hospital beds, clinics, and physicians was standardized per 1000 people, and the number of in-home service workers, day service workers, short-stay service workers, and long-term care facility workers was standardized per 1000 population 65 years or older. The levels of care needed were set at 7 levels by the local government, taking into account physical and cognitive functions. The most common underlying causes of death in the Japanese population 65 years or older (cancer, cerebrovascular diseases, cardiovascular diseases, senility, and pneumonia) were included. Marital status was categorized as present, unmarried, widow, or divorced. The place of death was whether death occurred at home or not.

At the municipal level, we considered *predisposing* characteristics, including the demographic characteristics (ie, population size, the proportion of the population ≥65 years of age, and the proportion of women ≥65 years of age), social characteristics reflecting the socioeconomic status (ie, the employment rate among adolescent girls and women 15-64 years of age, annual income per capita, and the proportion of school enrollment of those 15-19 years of age), and screening rates for colorectal cancer as a proxy for regional underlying health beliefs. We considered the following as *enabling* characteristics: municipal financial power index (ie, the 3-year mean of base financial revenue divided by base financial demand) and organizational factors to deliver health and LTC services, including the number of hospital beds, clinics, and physicians per 1000 people, the number of in-home service workers (ie, for care, nursing, and renting welfare equipment), day service workers (ie, for care and rehabilitation), short-stay service workers (ie, for care), and LTC facility workers (ie, at LTC welfare, health, and medical facilities, qualified nursing homes, and nursing homes for dementia patients) per 1000 population 65 years and older.^13^ Lastly, we considered the following as municipal *need* characteristics: environmental factors (ie, population density as a proxy of the degree of urbanization, the proportion of households with persons ≥65 years of age living alone, the proportion of households with older married couples with 1 man ≥65 years of age and 1 woman ≥60 years of age), and the mean life expectancy of both sexes as an index of the population health. The intentions behind the selection of these explanatory variables were detailed in the eMethods in the Supplement.

### Statistical Analysis

A 3-level logistic regression analysis was conducted to assess the extent to which contextual factors at the prefecture and municipality levels separately explain the variance in the place of death.^24,25^ Level 1 was the individual level (denoted as *i*), level 2 was the municipal level (*j*), and level 3 was the prefectural level (*k*). The probability (*p_i_*) of in-home death for the individual *i* can be written as follows:



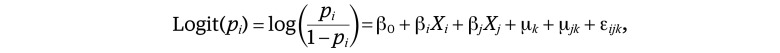



where *X_i_* and *X_j_* indicate the explanatory variables (fixed-effects variables) at the individual and municipal levels, respectively. *β_i_* and *β_j_* are coefficients, and *β_0_* is constant. *μ_k_* is a level 3 random intercept, *μ_jk_* is a level 2 random intercept, and *ε_ijk_* is a level 1 error residual.

We initially estimated the null (empty) model. In model 1, *X_i_* included the individual characteristics. In model 2, in addition to model 1, the *predisposing* contextual characteristics at the municipal level were added to *X_j_*. Model 3 next added the municipal *enabling* characteristics to *X_j_*. Finally, model 4 added the municipal *need* characteristics to *X_j_*.

We calculated the intraclass correlation coefficient (ICC) (also termed *variance partition coefficient*) in each model to understand the contribution of prefectures and municipalities to the variance of the place of death, using the following equations of the latent variable method^25^:



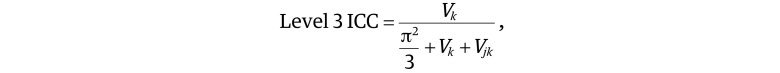





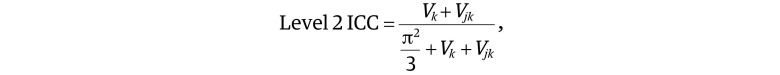



where *V_k_* is the level 3 variance and *V_jk_* is the level 2 variance. In addition, we show the median odds ratios (MORs) in each model to translate the prefectural- and municipal-level variances into the odds ratio scale, which has a consistent and intuitive interpretation, using the following equations^24,25^:



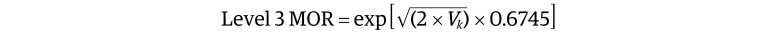





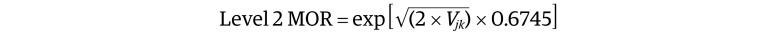



Furthermore, to evaluate the macrolevel variance changes, the percentage of proportional change in variance (PCV) was calculated as follows:



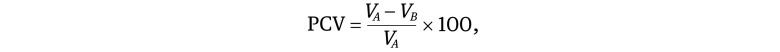



where *V_A_* is the variance of the initial model and *V_B_* is the variance of the model with more terms.^25^

All analyses were conducted using Stata software, version 16 MP (StataCorp LLC). Two-sided *P* < .05 was interpreted as statistically significant.

## Results

A total of 544 836 decedents (median [IQR] age, 87 [81-91] years; 300 142 [55.1%] female) were included in the study. The number of individuals represented 49.2% of all deaths of people 65 years or older, excluding external causes, in Japan during the study period. The remaining decedents did not apply to use the LTC insurance before death. Namely, they were in no need of LTC services before their death (eg, because of relatively acute-onset illness) or required mainly medical services under the health insurance system. For example, although the proportion of deaths from acute myocardial infarction was 2.2% and from cancer was 25.0% among those who used LTC services, the proportion of deaths from acute myocardial infarction was 3.5% and from cancer was 31.9% among those who did not use LTC services.

The proportion of home deaths (of all deaths) was 10.3% (55 990 decedents) (Table 1). Older people who died at home were more likely to have a lower degree of care needs (8.7% [4866 decedents] for in-home death vs 6.7% [32 383 decedents] for other places of death within the support levels), to have a spouse (44.2% [24 770 decedents] vs 38.7% [189 406 decedents]), and to have died of cancer, cardiovascular disease, or senility (33.9% [18 955 decedents] vs 23.4% [114 487 decedents] for cancer, 21.6% [12 121 decedents] vs 14.8% [72 587 decedents] for cardiovascular disease, and 15.0% [8391 decedents] vs 9.4% [45 752 decedents] for senility). The characteristics of the municipalities are provided in Table 2.

**Table 1.** Characteristics of Individual Factors by Place of Death^a^

Characteristic	Total	Home	Other places^b^
No. of deaths	544 836 (100)	55 990 (10.3)	488 846 (89.7)
Age, median (IQR), y	87 (81-91)	86 (79-92)	87 (81-91)
Sex			
Male	244 694 (44.9)	26 197 (46.8)	218 497 (44.7)
Female	300 142 (55.1)	29 793 (53.2)	270 349 (55.3)
Levels of care needed			
Support level			
1	16 737 (3.1)	2299 (4.1)	14 438 (3.0)
2	20 512 (3.8)	2567 (4.6)	17 945 (3.7)
Care level			
1	53 185 (9.8)	6296 (11.2)	46 889 (9.6)
2	72 515 (13.3)	8703 (15.5)	63 812 (13.1)
3	82 809 (15.2)	8232 (14.7)	74 577 (15.3)
4	132 490 (24.3)	11 730 (21.0)	120 760 (24.7)
5	166 588 (30.6)	16 163 (28.9)	150 425 (30.8)
Underlying cause of death			
Cancer	133 442 (24.5)	18 955 (33.9)	114 487 (23.4)
Cardiovascular disease	84 708 (15.5)	12 121 (21.6)	72 587 (14.8)
Pneumonia	66 032 (12.1)	1850 (3.3)	64 182 (13.1)
Senility	54 143 (9.9)	8391 (15.0)	45 752 (9.4)
Cerebrovascular disease	52 379 (9.6)	3783 (6.8)	48 596 (9.9)
Other	154 132 (28.3)	10 890 (19.4)	143 242 (29.3)
Marital status			
Present	214 176 (39.3)	24 770 (44.2)	189 406 (38.7)
Unmarried	20 719 (3.8)	1735 (3.1)	18 984 (3.9)
Widow	284 254 (52.2)	26 909 (48.1)	257 345 (52.6)
Divorce	25 687 (4.7)	2576 (4.6)	23 111 (4.7)

^a^ Data are presented as number (percentage) of individuals unless otherwise indicated.

^b^ Other places include hospitals, clinics, long-term care facilities, and other locations such as day service facilities or outdoors.

**Table 2.** Characteristics of the Municipalities

Characteristic	Median (IQR)
Proportion of in-home death, %	8.6 (5.2-12.3)
Population size per 1000 people	21.8 (7.9-55.1)
Proportion of the population ≥65 years of age, %	31.6 (26.9-36.6)
Proportion of women ≥65 years of age, %	57.1 (55.7-58.4)
Employment rate among adolescent girls and women 15-64 years of age, %	47.0 (43.9-49.7)
Annual income per capita (¥100 000)	26.8 (24.8-29.3)
Proportion of school enrollment of those who were 15-19 years of age, %	87.9 (85.8-90.0)
Screening rate of colorectal cancer, %	17.3 (11.5-27.0)
Financial power index (100-fold)	43 (25-68)
No. of hospital beds per 1000 people	9.5 (0-16.1)
No. of clinics per 1000 people	0.7 (0.5-0.9)
No. of physicians per 1000 people	1.3 (0.7-1.9)
No. of in-home service workers per 1000 population ≥65 years of age	5.4 (3.7-7.7)
No. of day service workers per 1000 population ≥65 years of age	10.7 (8.2-13.7)
No. of short-stay service workers per 1000 population ≥65 years of age	4.3 (2.3-7.3)
No. of long-term care facility workers per 1000 population ≥65 years of age	22.4 (17.3-28.9)
Population density, persons/100 m^2^	0.4 (0.2-0.9)
Proportion of households, %	
With older persons living alone	11.8 (9.4-15.4)
With older married couples	13.8 (11.6-16.4)
Mean life expectancy, y	
Men	80.6 (80.1-81.1)
Women	87.0 (86.6-87.3)

From the random-effects parameters in the multilevel logistic regression (Table 3), the intraclass correlation coefficients of the null model indicated that 7.2% of the variance in the place of death was attributable to municipal-level factors. Municipal characteristics were associated with more of the variance than were prefectural characteristics (2.7%). The largest proportional change (7.3%) in variance at the municipality level was observed when enabling factors (ie, medical and long-term care resources) were added to the model The MORs were 1.35 (95% CI, 1.31-1.38) for prefectures and 1.46 (95% CI, 1.42-1.51) for municipalities, indicating that variance attributable to municipal factors contributed more to the variance in the probability of in-home death compared with prefectural factors. The amount of proportional change in variance at the municipality level was the largest when going from model 2 (3.0%) to model 3 (10.3%) (ie, when *enabling* characteristics were added).

**Table 3.** Random-Effects Parameters in the Multilevel Logistic Regression

Random-effects parameter	Null model	Model 1	Model 2	Model 3	Model 4
**Prefectures level**					
Variances	0.10	0.09	0.06	0.06	0.05
SE	0.02	0.02	0.01	0.01	0.01
PCV, %	0	6.3	33.9	37.4	43.1
ICC	0.027	0.026	0.018	0.017	0.016
MOR	1.34	1.33	1.27	1.26	1.25
**Municipalities level**					
Variances	0.16	0.16	0.15	0.14	0.14
SE	0.02	0.02	0.02	0.02	0.02
PCV, %	0	0.4	3.0	10.3	11.9
ICC	0.072	0.070	0.062	0.058	0.056
MOR	1.46	1.46	1.46	1.43	1.43

Abbreviations: ICC, intraclass correlation coefficient; MOR, median odds ratio; PCV, proportional changes in variance.

The results of model 4 (Table 4) indicate that older people who died at home were more likely to have lower care needs, to die of cancer, cardiovascular disease, or senility, and to have a spouse. In addition, the municipalities with a higher proportion of in-home death tended to be more populous, to have a higher proportion of women 65 years or older, to have a higher supply of clinics, physicians, and in-home service workers per population, and to have a lower number of hospital beds and LTC facility workers per population.

**Table 4.** Odds Ratios of Explanatory Variables in the Multilevel Logistic Regression^a^

Variable	Odds ratio (95% CI)				
	Null model	Model 1	Model 2	Model 3	Model 4
Constant	0.10 (0.09-0.11)	0.09 (0.07-0.11)	0.67 (0.05-9.10)	0.76 (0.07-7.78)	0.07 (0-58.79)
Individual characteristics					
Age	NA	0.999 (0.996-1.002)	0.999 (0.996-1.002)	0.999 (0.996-1.002)	0.999 (0.996-1.002)
Sex					
Male	NA	1 [Reference]	1 [Reference]	1 [Reference]	1 [Reference]
Female	NA	0.983 (0.962-1.005)	0.983 (0.962-1.005)	0.983 (0.962-1.005)	0.983 (0.962-1.005)
Levels of care needed					
Support level					
1	NA	1.18 (1.13-1.25)	1.18 (1.13-1.25)	1.18 (1.13-1.25)	1.18 (1.13-1.25)
2	NA	1.12 (1.06-1.18)	1.12 (1.06-1.18)	1.12 (1.06-1.18)	1.12 (1.06-1.18)
Care level					
1	NA	1 [Reference]	1 [Reference]	1 [Reference]	1 [Reference]
2	NA	1.02 (0.98-1.07)	1.02 (0.98-1.07)	1.02 (0.98-1.07)	1.02 (0.98-1.07)
3	NA	0.88 (0.83-0.92)	0.88 (0.83-0.92)	0.87 (0.83-0.92)	0.87 (0.83-0.92)
4	NA	0.80 (0.74-0.85)	0.80 (0.74-0.85)	0.80 (0.74-0.85)	0.80 (0.74-0.85)
5	NA	0.91 (0.86-0.97)	0.91 (0.86-0.97)	0.91 (0.86-0.97)	0.91 (0.86-0.97)
Underlying cause of death					
Cancer	NA	2.03 (1.88-2.19)	2.03 (1.88-2.19)	2.03 (1.88-2.19)	2.03 (1.88-2.19)
Cerebrovascular	NA	1.05 (0.98-1.13)	1.05 (0.98-1.13)	1.05 (0.98-1.13)	1.05 (0.98-1.13)
Cardiovascular	NA	2.26 (2.06-2.49)	2.26 (2.06-2.49)	2.26 (2.06-2.49)	2.26 (2.06-2.49)
Pneumonia	NA	0.39 (0.36-0.42)	0.39 (0.36-0.42)	0.39 (0.36-0.42)	0.39 (0.36-0.42)
Senility	NA	2.59 (2.39-2.81)	2.59 (2.39-2.81)	2.59 (2.39-2.81)	2.59 (2.39-2.81)
Others	NA	1 [Reference]	1 [Reference]	1 [Reference]	1 [Reference]
Marital status					
Present	NA	1 [Reference]	1 [Reference]	1 [Reference]	1 [Reference]
Unmarried	NA	0.69 (0.63-0.75)	0.69 (0.63-0.75)	0.69 (0.63-0.75)	0.69 (0.63-0.75)
Widow	NA	0.80 (0.78-0.83)	0.80 (0.78-0.83)	0.80 (0.78-0.83)	0.80 (0.78-0.83)
Divorce	NA	0.83 (0.79-0.87)	0.83 (0.79-0.87)	0.83 (0.79-0.87)	0.83 (0.79-0.87)
Municipal characteristics					
Predisposing characteristics					
Population size	NA	NA	1.0001 (1.0000-1.0003)	1.0001 (1.0000-1.0002)	1.0001 (1.0000-1.0002)
Proportion of the population ≥65 years of age	NA	NA	1.00 (0.99-1.01)	1.00 (0.99-1.01)	1.02 (1.00-1.04)
Proportion of women ≥65 years of age	NA	NA	0.96 (0.93-0.98)	0.95 (0.93-0.98)	0.95 (0.91-1.00)
Employment rate among adolescent girls and women 15-64 years of age	NA	NA	1.00 (0.98-1.02)	1.00 (0.99-1.02)	0.99 (0.98-1.01)
Annual income per capita	NA	NA	1.01 (0.99-1.02)	1.00 (0.98-1.02)	1.00 (0.99-1.02)
Proportion of school enrollment of those who were 15-19 years of age	NA	NA	1.00 (0.99-1.01)	1.00 (0.99-1.01)	1.00 (0.99-1.01)
Screening rate of colorectal cancer	NA	NA	1.001 (0.998-1.003)	1.000 (0.998-1.003)	1.000 (0.998-1.002)
Enabling characteristics					
Financial power index	NA	NA	NA	0.999 (0.996-1.003)	0.999 (0.996-1.003)
No. of hospital beds	NA	NA	NA	0.990 (0.986-0.994)	0.990 (0.986-0.994)
No. of clinics	NA	NA	NA	1.12 (1.01-1.24)	1.13 (1.01-1.26)
No. of physicians	NA	NA	NA	1.04 (1.02-1.06)	1.04 (1.02-1.06)
No. of in-home service workers	NA	NA	NA	1.015 (1.003-1.028)	1.018 (1.005-1.031)
No. of day service workers	NA	NA	NA	1.008 (0.997-1.019)	1.007 (0.996-1.018)
No. of short-stay service workers	NA	NA	NA	0.998 (0.991-1.005)	0.999 (0.992-1.005)
No. of long-term care facility workers	NA	NA	NA	0.996 (0.992-1.001)	0.995 (0.991-1.000)
Need characteristics					
Population density	NA	NA	NA	NA	1.00 (0.96-1.05)
Proportion of households with older persons living alone	NA	NA	NA	NA	0.98 (0.95-1.01)
Proportion of households with older married couples	NA	NA	NA	NA	0.972 (0.940-1.005)
Mean life expectancy					
Men	NA	NA	NA	NA	1.04 (0.97-1.11)
Women	NA	NA	NA	NA	1.00 (0.93-1.08)

Abbreviation: NA, not applicable.

^a^ All models included random intercepts for prefecture and municipality. The number of digits after the decimal point was expressed to enable the identification of statistical significance from the 95% CI.

## Discussion

This cross-sectional study explored the potential associations of municipal-level characteristics with the variation in in-home death of care-dependent older people using 3-level logistic regression analysis. Our analysis revealed substantial (7.2%) municipal variation in in-home deaths among care-dependent older people. Furthermore, our results showed that municipality-level enabling characteristics had more influence on the variation in the place of death than the prefecture-level enabling characteristics. Stated differently, our results suggest that to create an environment in which care-dependent older people can stay at home at the end of their lives, municipal policy makers need to focus on securing an adequate supply of clinics and physicians attending to end-of-life care, in addition to increasing the supply of in-home service workers.

Previous ecological studies^14,15,16,17,18^ have reported a positive association between good access to in-home services provided by physicians, nurses, and care workers and in-home death. By contrast, high accessibility to hospital beds and LTC facilities has been associated with a lower likelihood of in-home death (as we found in the current study).^15,16,17,18^ Although it is possible that some patients substitute one service for another (eg, some patients might be forced to die at home when there is insufficient local hospital bed supply), our interpretation is that there are more patients who miss out on dying at home because of an insufficient supply of physicians and care workers who are able to provide domiciliary end-of-life care. Because Japan has far more hospital beds per capita than other high-income countries, people may spend more of their final days in hospitals.^26^

Increasing the number of clinics, physicians, and care workers for in-home services per population in a municipality would mean that residents would have easier access to those services. In a previous study^12^ that examined the association between the use of in-home care services and in-home death, residents in municipalities with more care workers used the domiciliary services at a higher rate. In Japan, when considering where to die, 73% of older persons are concerned about the potential burden of care on their families, followed by 57% who cite being able to live without physical and psychological symptoms as a condition.^9^ The use of in-home services provided by physicians, nurses, and care workers would have helped reduce the burden of caregiving on informal caregivers and help care-dependent older adults feel more confident and secure in their home care and stabilize their symptoms.^4,27,28^

Our findings provide several implications for policy makers when planning regional medical care and LTC. In Japan, the end-of-life care of care-dependent older adults at home is traditionally charged to the family members to a large extent. However, our results suggest that municipal policy makers could assist older residents and their families by coordinating local medical and LTC resources. Another implication of our study is that when planning local medical and LTC services, authorities might consider increasing the supply of clinics, physicians, and care workers focused on delivering in-home services, as opposed to further expanding the supply of hospital beds and LTC facility workers. Considering the current situation in which more than half of older persons wish to die at home but only 10% have their wish fulfilled, these policies could satisfy the preferences of care-dependent older people at the end of life.

### Limitations

This study has several limitations. First, some individuals were excluded from the analysis because of duplicate identifiers and missing values, which may have led to sampling bias. Nevertheless, our analysis includes 95% of the individuals from all over Japan, and we believe it is unlikely that our conclusions have been significantly distorted. Second, our study design is cross-sectional; hence, a causal relationship between the explanatory variables and the dependent variable cannot be determined. Third, there may be unmeasured confounders, such as the older persons’ and families’ preferences for the place of death. Fourth, the place of death is a proxy for the quality of dying and death. To assess the association of the quality of dying and death with municipal characteristics, use of more comprehensive measurement tools for the quality of dying and death would be needed.^3^

## Conclusions

This cross-sectional study found considerable contextual variation in the place of death, which is explained by individual factors and municipal characteristics. These results suggest that policy makers need to ensure an adequate supply of clinics, physicians, and care workers for in-home services to meet the preferences of care-dependent older people who wish to spend their final days at home. Further research is needed to determine whether changes in these supplies alter the probability of in-home death.

## Notes

Supplement. **eMethods.** Selection of Explanatory Variables **eTable.** Summary of Data Sources for Each Variable
