# Supplementary material for: Municipal Characteristics of In-Home Death Among Care-Dependent Older Japanese Adults
Source: JAMA Netw Open. 2022 Jan 5;5(1):e2142273. doi: 10.1001/jamanetworkopen.2021.42273 (PMC8733841; doi:10.1001/jamanetworkopen.2021.42273)
Supplement: Supplement. — eMethods. Selection of Explanatory Variables eTable. Summary of Data Sources for Each Variable [file jamanetwopen-e2142273-s001.pdf]

Supplementary Online Content

Abe K, Kawachi I, Taniguchi Y, Tamiya N. Municipal characteristics of in-home death among care-dependent older Japanese adults. *JAMA Netw Open*. 2022;5(1):e2142273. doi:10.1001/jamanetworkopen.2021.42273

eMethods. Selection of Explanatory Variables

eTable. Summary of Data Sources for Each Variable

This supplementary material has been provided by the authors to give readers additional information about their work.

© 2022 Abe K et al. *JAMA Network Open.*

eMethods. Selection of Explanatory Variables

Based on previous studies as well as the availability of data, the patients' age at death, sex, level of care needed, the underlying causes of death, and marital status were included as individual characteristics in the models. The level of care needed was added as an indicator of the physical and cognitive functions of the patient. The marital status was used as a proxy for the availability of informal caregivers at home. We predicted that older people who die at home are more likely to have a spouse, have lower care needs, and die of cancer, cardiovascular disease, senility, and cerebrovascular disease.

In addition, at the municipal level, the population size, the proportion of the population ≥65 years of age, and the proportion of women ≥65 years of age were included as demographic characteristics. The proportion of women ≥65 years of age might be correlated with the extent to which long-term care services are needed, since older women are more likely to be widowed and live alone due to differences in mean life expectancy. The employment rate among adolescent girls and women 15-64 years of age, annual income per capita, the proportion of school enrollment of those who were 15-19 years of age were considered as indicators of the socioeconomic status of the municipalities. Among them, the employment rate among adolescent girls and women 15-64 years of age might reflect the presence of an informal caregiver at home because women have traditionally been in charge of caregiving in Japan. Screening rates for colorectal cancer were considered as a proxy of health beliefs underlying the region. The financial power index indicates the financial capacity, and the number of hospital beds, clinics, physicians, in-home service workers, short-stay service workers, and long-term care facility workers per population reflects the accessibility of medical and long-term care services in the municipality. The population density was used as a proxy for the degree of urbanization, and the proportion of households with older persons living alone and the proportion of households with older married couples were used as indicators that reflect the needs of long-term care services. The mean life expectancy of both sexes was used as an index of population health. We predicted that the following characteristics are positively correlated with in-home deaths: population size, the proportion of the population ≥65 years of age, annual income per capita, the proportion of school enrollment of those who were 15-19 years of age, screening rate of colorectal cancer, financial power index, the number of clinics, physicians, in-home service workers, day service workers, and short-stay workers per population, population density, and the mean life expectancy. By contrast, we predicted that the following characteristics are inversely correlated with in-home deaths: the proportion of women ≥65 years of age, the employment rate among adolescent girls and women 15-64 years of age, the number of hospital beds and long-term care facility workers, the proportion of households with older persons living alone, and the proportion of households with older married couples.

© 2022 Abe K et al. *JAMA Network Open.*

eTable. Summary of Data Sources For Each Variable

Variables	Data sources
**Individual characteristics**	
**Age**	Vital Statistics in 2015
**Sex**	
**Marital status**	
**Place of death**	
**Underlying cause of death**	
**Levels of care needed**	Statistics of Long-term Care Benefit Expenditures in 2015
**Contextual characteristics**	
**Population size**	Population Census in 2015
**Proportion of the population ≥65 years of age**	
**Proportion of women ≥65 years of age**	
**Employment rate among adolescent girls and women 15-64 years of age**	
**Annual income per capita**	Survey of Municipal Taxation in 2015
**Proportion of school enrollment of those who were 15-19 years of age**	Population Census in 2010
**Screening rate of colorectal cancer**	Comprehensive Survey of Living Conditions in 2015
**Financial power index**	Annual Statistics on Local Public Finance in 2015
**No. of hospital beds**	Survey of Medical Institutions in 2015
**No. of clinics**	
**No. of physicians**	Statistics of Physicians, Dentists and Pharmacists in 2014
**No. of in-home service workers**	Survey of Institutions and Establishments for Long-term Care in 2015
**No. of day service workers**	
**No. of short-stay service workers**	
**No. of long-term care facility workers**	

© 2022 Abe K et al. *JAMA Network Open.*

**Population density**	Population Census and Statistical reports on land areas in 2015
**Proportion of households with older persons living alone**	Population Census in 2015
**Proportion of households with older married couples**	
**Mean life expectancy**	Life Tables in 2015

© 2022 Abe K et al. *JAMA Network Open.*
